# Enhanced Photodynamic Anticancer Activities of Multifunctional Magnetic Nanoparticles (Fe_3_O_4_) Conjugated with Chlorin e6 and Folic Acid in Prostate and Breast Cancer Cells

**DOI:** 10.3390/nano8090722

**Published:** 2018-09-13

**Authors:** Kyong-Hoon Choi, Ki Chang Nam, Guangsup Cho, Jin-Seung Jung, Bong Joo Park

**Affiliations:** 1 Institute of Biomaterials, Kwangwoon University, Nowon-gu, Seoul 01897, Korea; solidchem@hanmail.net; 2 Department of Medical Engineering, Dongguk University College of Medicine, Gyeonggi-do 10326, Korea; kichang.nam@gmail.com; 3 Department of Electrical & Biological Physics, Kwangwoon University, Nowon-gu, Seoul 01897, Korea; gscho@kw.ac.kr; 4 Department of Chemistry, Gangneung-Wonju National University, Gangneung 25457, Korea

**Keywords:** multifunctional magnetic nanoparticles, chlorin e6, folic acid, in vivo penetration depth, cancer cell targeting

## Abstract

Photodynamic therapy (PDT) is a promising alternative to conventional cancer treatment methods. Nonetheless, improvement of in vivo light penetration and cancer cell-targeting efficiency remain major challenges in clinical photodynamic therapy. This study aimed to develop multifunctional magnetic nanoparticles conjugated with a photosensitizer (PS) and cancer-targeting molecules via a simple surface modification process for PDT. To selectively target cancer cells and PDT functionality, core magnetic (Fe_3_O_4_) nanoparticles were covalently bound with chlorin e6 (Ce6) as a PS and folic acid (FA). When irradiated with a 660-nm long-wavelength light source, the Fe_3_O_4_-Ce6-FA nanoparticles with good biocompatibility exerted marked anticancer effects via apoptosis, as confirmed by analyzing the translocation of the plasma membrane, nuclear fragmentation, activities of caspase-3/7 in prostate (PC-3) and breast (MCF-7) cancer cells. Ce6, used herein as a PS, is thus more useful for PDT because of its ability to produce a high singlet oxygen quantum yield, which is owed to deep penetration by virtue of its long-wavelength absorption band; however, further in vivo studies are required to verify its biological effects for clinical applications.

## 1. Introduction

Cancer is a leading cause of mortality worldwide. Every year, an estimated 11 million individuals are diagnosed with cancer, and approximately 7 million individuals die of cancer according to the World Health Organization (WHO) [1]. Therefore, cancer currently ranks among the deadliest diseases, and advancements in medical technology have yielded various methods for cancer treatment over the last few decades [2]. Among them, traditional chemotherapy is limited by its severe toxicity, poor tumor-specific delivery, and the possibility of inducing multi-drug resistance [3,4,5]. However, in comparison with chemotherapy, photodynamic therapy (PDT) offers certain unique advantages including minimal invasiveness, fewer side effects, negligible chemotherapeutic resistance, and low systematic toxicity [6,7,8].

In PDT, photosensitizers (PS) are the key components that transfer photo-energy to the surrounding O_2_ molecules, generating reactive oxygen species (ROS), primarily singlet oxygen (^1^O_2_), to eliminate proximal cancer cells [9,10,11,12]. According to a recent study, various PSs have developed, which absorb light over a broad range from ultraviolet (UV) to the near-infrared (NIR) range [13,14]. However, the absorption bands of many PSs are primarily in the UV-Vis region [15,16]. Furthermore, the PSs with the absorption band in the NIR range have low ^1^O_2_ quantum yield owing to a low population of PSs in the triplet state [17,18]. Therefore, these PSs are often limited by their low ^1^O_2_ quantum yields, and low depth of penetration resulting from a short excitation wavelength [15,16]. In addition, currently available PSs are mostly nonspecifically activated and have poor water solubility and stability and low accumulation at the target site, resulting in treatment-related toxicity, light-induced degradation of drug molecules, and other side effects on adjacent normal tissue and blood cells [6,19,20,21]. To overcome these limitations, various inorganic and organic nanocarriers, including Fe_3_O_4_ nanoclusters, Au nanoparticles, graphene oxide, mesoporous silica nanoparticles, and polymer micelles, have been used to improve the stability and therapeutic outcomes of these PSs [22,23,24,25,26]. Nonetheless, the development of multifunctional nanoparticles with enhanced anticancer efficiency remains a major challenge in PDT.

Herein, to achieve enhanced photodynamic anticancer activity, we designed and fabricated a novel Fe_3_O_4_ nanoparticle conjugated with chlorin e6 (Ce6) and folic acid (FA) (Fe_3_O_4_-Ce6-FA) via simple surface modification. To enhance the PDT efficiency, magnetic core particles were conjugated with Ce6 and FA as PDT agents to increase the in vivo penetration depth of the light source and selectively eliminate cancer cells. In addition, we evaluated the efficiency of the Fe_3_O_4_-Ce6-FA nanoparticles for specific targeting and photodynamic anticancer activity in vitro. The Fe_3_O_4_-Ce6-FA nanoparticles developed herein could be a promising multifunctional nanoreagent for photodynamic tumor therapy and multifunctional drug delivery in the future.

## 2. Results and Discussion

### 2.1. Characterization of Multifunctional Fe_3_O_4_-Ce6-FA

**Scheme 1:**
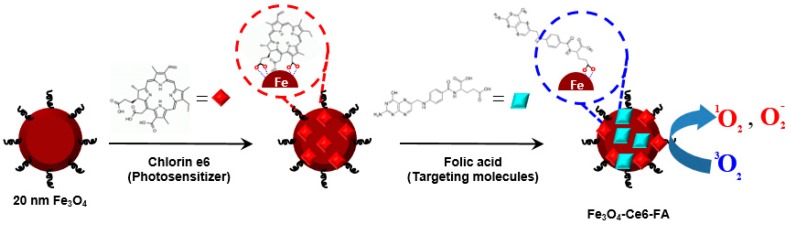
Fabrication procedure for the multifunctional magnetic nanoparticles.

Multifunctional 20-nm Fe_3_O_4_-Ce6-FA nanoparticles were fabricated via a simple surface modification via a wet chemical process as shown in Scheme 1. Ce6, having a long-wavelength absorption band and high singlet oxygen quantum yield, was conjugated with Fe ions on the surface of the Fe_3_O_4_ nanoparticles via esterification. Additionally, the multifunctional nanoparticles were functionalized with FA used as a targeting molecule to deliver these particles to the cancer cell membrane.

**Figure 1:**
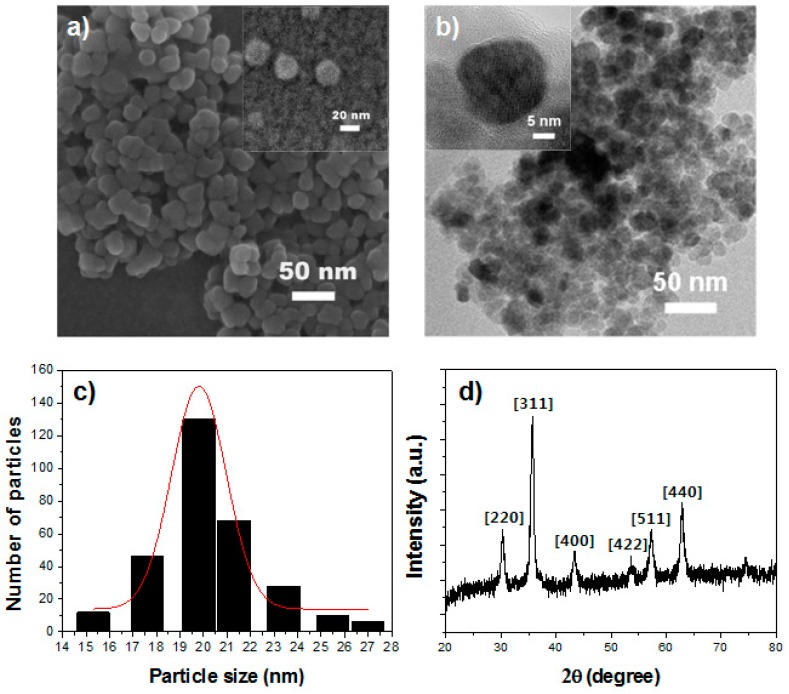
Structural analysis of the cholrin6- and folic acid-conjugated magnetite (Fe_3_O_4_-Ce6-FA) nanoparticles. (**a**) Field emission scanning electron and (**b**) transmission electron micrographs of the Fe_3_O_4_-Ce6-FA nanoparticles; (**c**) histogram of particle size distribution; (**d**) X-ray diffraction pattern of the Fe_3_O_4_-Ce6-FA nanoparticles.

Transmission electron microscopy (TEM), field emission scanning electron microscopy (FE-SEM), and X-ray diffraction (XRD) analysis were performed to confirm the appearance, size distribution, and crystallinity of the Fe_3_O_4_-Ce6-FA nanoparticles. As shown in Figure 1a,b, Fe_3_O_4_-Ce6-FA nanoparticles had a uniform spherical structure and a rough surface, measuring approximately 20 nm in diameter (Figure 1a inset). High-resolution TEM (HRTEM) images revealed regular parallel lattice fringes, indicating the high crystallinity of the Fe_3_O_4_-Ce6-FA nanoparticles (inset of Figure 1b). The lattice spacing of 0.26 nm was consistent with the in-plane lattice spacing of the (311) planes in the typical spinel structure of magnetite nanoparticles. The size histogram indicates that the average size of the particles was 19.8 nm with a distribution of 1.16 nm (Figure 1c). The wide-angle XRD pattern of Fe_3_O_4_-Ce6-FA nanoparticles can be indexed to the typical cubic structure of spinel Fe_3_O_4_ (JCPDS No. 19-629). The six strong Bragg reflection peaks (2θ = 30.2°, 35.7°, 43.4°, 53.6°, 57.4°, 63.0°), marked by their Miller indices ((220), (311), (400), (422), (511), and (440)) were obtained from standard Fe_3_O_4_ powder diffraction data (Figure 1d).

**Figure 2:**
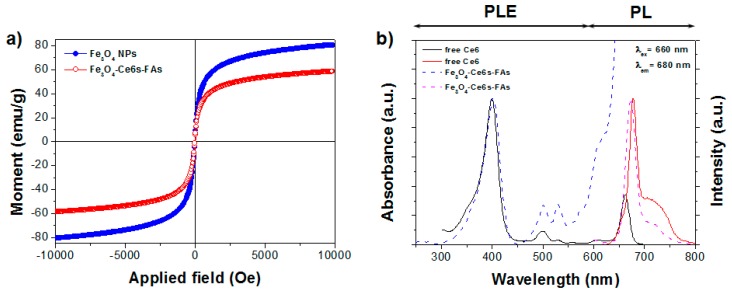
Magnetic and optical properties of the cholrin6- and folic acid-conjugated magnetite (Fe_3_O_4_-Ce6-FA) nanoparticles. (**a**) Room temperature magnetic hysteresis loops of pure Fe_3_O_4_ and Fe_3_O_4_-Ce6-FA nanoparticles; (**b**) Photoluminescence and photoluminescence excitation spectra of free Ce6 and the Fe_3_O_4_-Ce6-FA in THF.

Figure 2a shows the magnetic hysteresis loops of pure Fe_3_O_4_ and Fe_3_O_4_-Ce6-FA nanoparticles at room temperature. As shown, both samples exhibited superparamagnetic behavior without obvious remnant magnetization and coercivity owing to their small magnetite nanocrystal composition. The saturation magnetization (Ms) of pure Fe_3_O_4_ was 80.5 emu/g. After coating with PS and FA, the Ms of Fe_3_O_4_-Ce6-FA nanoparticles decreased to 58.5 emu/g. The minor reduction in magnetization primarily resulted from the reduction in the density of Fe_3_O_4_ due to the presence of non-magnetic coating layers. However, the Fe_3_O_4_-Ce6-FA nanoparticles (20 nm) still showed strong magnetization, thereby suggesting their suitability for magnetic separation and magnetic resonance (MR) imaging.

Figure 2b shows the photoluminescence (PL) and photoluminescence excitation (PLE) spectra of the pure Ce6 and the Fe_3_O_4_-Ce6-FA nanoparticles in THF. Ce6 displayed three main UV-Vis absorption peaks with an intense Soret band at 400 nm and two relatively weak Q-bands at 500 and 662 nm, respectively. After encapsulation of the Fe_3_O_4_ nanoparticles, a remarkable red shift and peak broadening in the UV-Vis spectrum of Fe_3_O_4_-Ce6-FA nanoparticles were observed, indicating the strong bonding between Ce6 and the magnetite nanoparticle [27]. Upon excitation at 660 nm, free Ce6 exhibited two strong emission peaks at 672 and 707 nm. The Fe_3_O_4_-Ce6-FA nanoparticles also exhibited a red shift and broadening compared with free Ce6, concurrent with the phenomenon observed during absorption.

**Figure 3:**
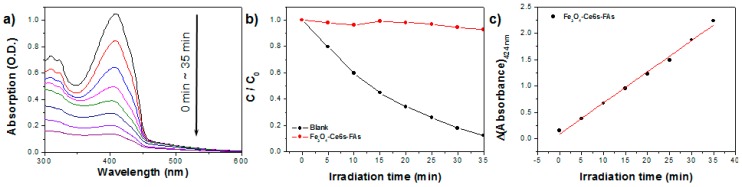
(**a**) UV-Vis spectra of 1,3-diphenylisobenzofuran (DPBF) in ethanol with the chlorin e6- and folic acid-conjugated magnetite (Fe_3_O_4_-Ce6-FA) nanoparticles in accordance with the irradiation time with a red LED lamp (λ_max_ = 660 nm); (**b**) The kinetic curve of the photodegradation efficiency of DPBF as a function of irradiation time; (**c**) Comparison of first-order degradation rates of DPBF.

We used 1,3-diphenylisobenzofuran (DPBF), a specific ^1^O_2_ probe, to quantify the ^1^O_2_ generated from the Fe_3_O_4_-Ce6-FA nanoparticles by monitoring the absorbance of DPBF at 424 nm. Figure 3a exhibits the time-dependent UV-Vis absorption spectra of complexes of DPBF and Fe_3_O_4_-Ce6-FA in ethanol, which were irradiated with a red light-emitting diode (LED) light source. Upon excitation at 660 nm, the intensity of the absorbance peak of DPBF at 424 nm decreased gradually with the increase in irradiation time in the presence of the Fe_3_O_4_-Ce6-FA nanoparticles (Figure 3b). In the blank condition, no appreciable degradation of DPBF was observed after irradiation for 35 min. Near-complete photodegradation of DPBF in the presence of Fe_3_O_4_-Ce6-FA nanoparticles was observed within 35 min. This clearly indicates that the Fe_3_O_4_-Ce6-FA nanoparticles can effectively generate the ^1^O_2_ ROS. Based on the reaction kinetics, which was well fitted into the equation In(C/C_0_) = −*k_obs_* × time (min), the apparent first-order rate constant, *k_obs_*, of DPBF photo-oxidation was 0.05094 min^−1^ for the Fe_3_O_4_-Ce6-FA nanoparticles (Figure 3c).

### 2.2. In Vitro Cytotoxicity of Multifunctional Fe_3_O_4_-Ce6-FA Nanoparticles

**Figure 4:**
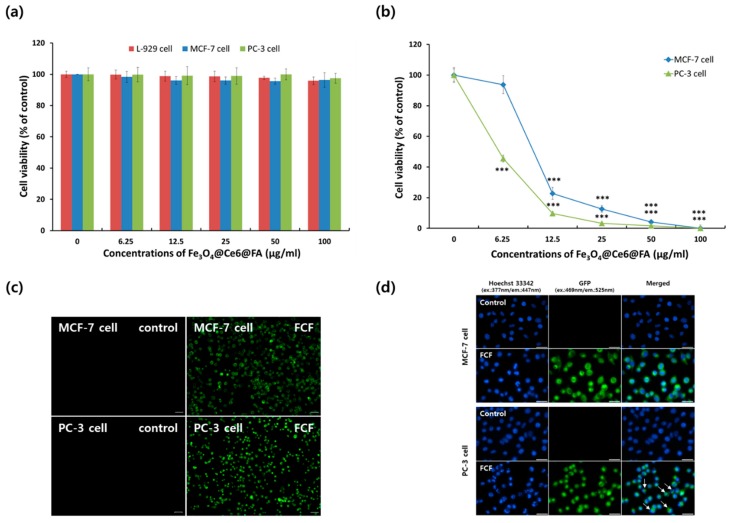
Biocompatibility and photodynamic anticancer activities of chlorin e6 and folic acid-conjugated magnetite (Fe_3_O_4_-Ce6-FA) nanoparticles. (**a**) Cytotoxicity and (**b**) phototoxicity of Fe_3_O_4_-Ce6-FA nanoparticles in MCF-7 (breast adenocarcinoma) and PC-3 (prostate adenocarcinoma) cell lines. Quantitative data are expressed as the mean ± standard deviation (*n* = 4), and the statistical comparisons were evaluated using Student’s *t*-test. Significant differences were indicated by *p* < 0.05 (*** *p* < 0.0005 vs. control). (**c**) Images of MCF-7 and PC-3 cells after staining with fluorescein isothiocyanate-conjugated Annexin V (Annexin V-FITC) thus demonstrating the membrane translocation of the cells. The green fluorescence signal was produced by Annexin V-FITC. “FCF” represents Fe_3_O_4_-Ce6-FA nanoparticles. Scale bar = 50 μm. (**d**) Nuclear fragmentation and caspase-3/7 activity in MCF-7 and PC-3 cells. The cells were stained with Hoechst 33342 to detect nuclear fragmentation and CellEvent Caspase-3/7 Green Detection reagent to detect caspase-3/7 activity after 6 h post photodynamic therapy at 20 mW for 30 min. Arrows represent apoptotic bodies of cells. Scale bar= 30 μm.

For biomaterials to be used for biomedical applications, a basic biocompatibility assay is necessary to evaluate their cytotoxicity. Therefore, the in vitro cytotoxicity of Fe_3_O_4_-Ce6-FA was evaluated in normal fibroblast (L-929), breast cancer (MCF-7), and prostate cancer (PC-3) cell lines, as described previously [28,29,30,31,32,33]. Twofold-diluted concentrations of Fe_3_O_4_-Ce6-FA from 100 to 6.25 μg/mL were tested, and non-treated cells constituted the control. As shown in Figure 4a, the cell viabilities of all cells exceeded 95%, indicating that Fe_3_O_4_-Ce6-FA displayed no cytotoxicity in all cells, suggesting that Fe_3_O_4_-Ce6-FA nanoparticles may have biomedical applications with excellent biocompatibility.

### 2.3. In Vitro Photodynamic Anticancer Activity of Fe_3_O_4_-Ce6-FA Nanoparticles

To confirm the photo-killing ability of Fe_3_O_4_-Ce6-FA nanoparticles, PC-3 and MCF-7 cell lines were exposed to LED irradiation for 10 min after incubation with various concentrations of Fe_3_O_4_-Ce6-FA nanoparticles for 2 h. As shown in Figure 4b, cell viabilities of the two cell lines were significantly decreased with an increase in nanoparticle concentration, and the cell viability of PC-3 cells was even more drastically decreased compared to that of MCF-7 cells, even at the lowest concentration of 6.25 μg/mL. This indicated that the photo-killing efficacy of Fe_3_O_4_-Ce6-FA nanoparticles was concentration-dependent. Moreover, Fe_3_O_4_-Ce6-FA nanoparticles are more effective than Fe_3_O_4_ conjugated with hematoporphyrins (HPs) and FA, as reported previously [30,31]. The PS (Ce6) used herein is more applicable for PDT owing to its attributes, which include a high singlet oxygen quantum yield and long-wavelength absorption band, resulting in deeper light penetration in vivo compared with HP-conjugated nanoparticles. In other words, the photodynamic anticancer efficacy of Fe_3_O_4_-Ce6-FA nanoparticles was closely associated with singlet oxygen quantum yield and the concentration of the Fe_3_O_4_-Ce6-FA nanoparticles.

Considering the photodynamic anticancer activity of Fe_3_O_4_-Ce6-FA nanoparticles, the mechanisms underlying cancer cell death were evaluated via analysis of the translocation of the plasma membrane, using an Annexin V-fluorescein isothiocyanate (FITC) apoptosis detection kit, nuclear fragmentation using a fluorescent dye (Hoechst 33342), and enzyme activities of caspase-3/7 using a CellEvent Caspase-3/7 Green Detection reagent. First, PC-3 and MCF-7 cells were stained with Annexin V-FITC reagent post-irradiation after incubation with Fe_3_O_4_-Ce6-FA nanoparticles for 2 h to confirm phosphatidylserine translocation from the intracellular to the extracellular leaflet of the plasma membrane, which is a hallmark of the early stage of apoptotic cell death. Figure 4c shows the images of live and apoptotic cells stained with Annexin V-FITC, post-irradiation. Both cell types (MCF-7 and PC-3) in the Fe_3_O_4_-Ce6-FA nanoparticle-treated groups showed green fluorescence, whereas control cells did not. These results indicate that PDT following treatment with Fe_3_O_4_-Ce6-FA nanoparticles induced cancer cell death via apoptosis.

Additionally, nuclear fragmentation of cancer cells, which is also a hallmark of apoptotic cell death, was confirmed via staining with Hoechst 33342 dye. As shown in Figure 4d, the nuclei of both cell types treated with Fe_3_O_4_-Ce6-FA nanoparticles were more condensed than those of control cells, and most nuclei of PC-3 cells rapidly changed to granular apoptotic nuclear bodies. However, no changes were detected in the control cells of both cell lines. These results also indicated that irradiation after treatment with Fe_3_O_4_-Ce6-FA nanoparticles enhanced apoptotic cell death.

Finally, caspase-3/7 activity, which essentially contributes to apoptotic cell death, were also evaluated using a fluorogenic substrate highly specific for activated caspase-3 and -7. As shown in Figure 4d, both cell types (MCF-7 and PC-3) treated with Fe_3_O_4_-Ce6-FA nanoparticles showed strong green fluorescence. Moreover, the PC-3 cells treated with Fe_3_O_4_-Ce6-FA nanoparticles showed morphological changes following irradiation, as indicated by the presence of apoptotic cellular bodies. These results indicate that cells treated with Fe_3_O_4_-Ce6-FA nanoparticles expressed high amounts of activated caspase-3 and -7 upon irradiation, and that the cells underwent apoptotic cell death. Overall, the results indicate that Fe_3_O_4_-Ce6-FA nanoparticles induced apoptotic cell death.

## 3. Materials and Methods

### 3.1. Preparation of Fe_3_O_4_-Ce6-FA Nanoparticles

Multifunctional Fe_3_O_4_-Ce6-FA nanocomposites were synthesized in accordance with a previously reported procedure with minor modifications [28]. In brief, FeCl_3_·6H_2_O (0.54 g) and NaAc·3H_2_O (1.5 g) in 20 mL ethylene glycol (EG) and diethylene glycol (DEG) (1:19) were added in a 200 mL round-bottom flask, and the mixture was vigorously stirred for 30 min. Thereafter, the yellowish homogeneous solution formed was sealed in a teflon-lined stainless steel autoclave. The autoclave was heated to and maintained at 200 °C for 10 h and cooled to ambient temperature. The black precipitate was harvested via magnetic decantation, washed with deionized water and absolute alcohol several times, and then dried in a vacuum oven at 60 °C for 12 h. The photoactive and targeting functionalities of the Fe_3_O_4_ nanoparticles were achieved using a wet chemical process similar to our previous method [29]. Briefly, 20 mg of precipitated Fe_3_O_4_ nanoparticles with 20 nm size were mixed with a solution of Ce6/EtOH (final concentration, 10^−4^ M). The Ce6 molecules are easily conjugated to the surface of magnetite nanoparticles owing to the three terminal carboxyl groups, which initiate covalent bonding. Furthermore, the Ce6 molecules have a high singlet oxygen quantum yield of 0.77 in solution [34]. This value of singlet oxygen quantum yield is higher than that of the other photosensitizers such as hematoporphyrin (0.51) [35] and protoporphyrin (0.63) [36]. The solution was vigorously agitated for 24 h at room temperature. After the reaction was completed, the product was washed several times with EtOH. To facilitate targeting functionality, the FA molecules were conjugated to Ce6-bonded Fe_3_O_4_ nanoparticles (Fe_3_O_4_-Ce6). Similarly, the washed Fe_3_O_4_-Ce6 nanoparticles were fully dispersed in 20 mL of FA/dimethylsulfoxide (DMSO) solution (3.7 × 10^−4^ M). The mixture solution was stirred for another 5 h at 25 °C. The Fe_3_O_4_-Ce6-FA nanocomposites were separated via magnetic decantation and washed with DMSO and phosphate-buffered saline (PBS; pH = 7.2) several times. Thereafter, the resulting nanoparticles were finally dried in vacuum at room temperature for 24 h. The concentration of HP and FA bonded to the surfaces of the Fe_3_O_4_ nanoparticles was estimated using UV-Vis absorption spectroscopy.

### 3.2. Physical Characterization of Multifunctional Fe_3_O_4_-Ce6-FA Particles

TEM (JEM-2100F, JEOL, Tokyo, Japan) and FE-SEM (SU-70, Hitachi, Tokyo, Japan) were performed to study the morphology of the multifunctional nanoparticles. The crystallographic structure of the composite particles was investigated via XRD (X’Pert Pro MPD, PANalytical, Almelo, Netherlands), using Cu Kα radiation. A vibrating sample magnetometer (VSM; Lakeshore 7300, Lake Shore Cryotronics, Westerville, OH, USA) was used to obtain magnetization versus magnetic field loop up to H = 10 kOe at room temperature. Steady-state absorption and PL and PLE spectra were measured using a UV–Vis spectrophotometer (U-2800, Hitachi, Tokyo, Japan) and spectrofluorometer (F-4500, Hitachi, Tokyo, Japan), respectively.

### 3.3. Biocompatibility of Fe_3_O_4_-Ce6-FA Nanoparticles

Cytotoxicity of the Fe_3_O_4_-Ce6-FA (FCF) nanoparticles was evaluated in L-929 (mouse fibroblasts), MCF-7 (breast adenocarcinoma), and PC-3 (prostate adenocarcinoma) cell lines, as described previously [28,29,30,31,32,33]. Briefly, all cells were seeded in a 24-well plate at 1.5 × 10^5^ cells/mL and incubated at 37 °C in 5% CO_2_ for 24 h, followed by further incubation with various concentrations (0, 6.25, 12.5, 25, 50, and 100 μg/mL) of FCF nanoparticles after exchanging the media with fresh media, in the dark. After another 24 h incubation, the viable cells were quantified using Cell Counting Kit-8 (CCK-8; Dojindo Laboratories, Kumamoto, Japan) assay reagent in accordance with the manufacturer’s instructions after washing three times with Dulbecco’s phosphate-buffered saline (DPBS). The optical density for each well was measured using a multimode microplate reader (Cytation 3, BioTek Instruments, Inc., Winooski, VT, USA) with an optical filter at 450 nm, and cell viability was determined in comparison with the untreated control.

### 3.4. Photodynamic Anticancer Activity of Multifunctional FCF Nanoparticles

Anticancer activity of the FCF nanoparticles was also assessed in MCF-7 and PC-3 cells on the basis of cell viability determined using CCK-8 after irradiation, as described previously [33,34,35,36]. Each cell type was plated in a 24-well plate at the same concentration and incubated as described in 3.3. Thereafter, the cells were further incubated with various concentrations (0, 6.25, 12.5, 25, 50, and 100 μg/mL) of FCF nanoparticles for 2 h in the dark, followed by three washes with DPBS, replenishment of the media, and irradiation with a red light-emitting diode (LED; FD-332R-N1, Fedy Technology Co., Shenzhen, China). The LEDs were driven using a constant current buck driver (LED-2800, TMC Co., Gunpo, Korea) and light intensity was regulated via pulse width modulation (CB210, Comfile Technology, Seoul, Korea). LED irradiation was applied at a maximum wavelength of 660 nm at 20 mW/cm^2^. After irradiation for 30 min, cancer cells were further incubated for 24 h, and cell viability was determined via a CCK-8 assay, as described in Section 3.3.

To evaluate the mechanisms underlying cancer cell death by Fe_3_O_4_-Ce6-FA after irradiation, both cancer cell types pre-cultured for 24 h were further incubated with 12.5 μg/mL of FCF after replenishing media in the dark. After 2 h of incubation, each cell type was irradiated with LED light at the same power for 10 min, as described previously in this section, and further incubated for 6 h to induce cell death. Thereafter, the plasma membranes and nuclei of both cell types were stained with an Annexin V-FITC apoptosis detection reagent (Komabiotech Inc., Seoul, Korea), Hoechst 33342 dye (Invitrogen, Molecular Probes, Eugene, OR, USA), and a CellEvent Caspase-3/7 Green Detection reagent (Invitrogen) in accordance with the manufacturers’ instructions. After staining each sample, fluorescence microscopic images were acquired using an automated live cell imager (Lionheart FX; BioTek Instruments, Inc., VT, USA).

## 4. Conclusions

In summary, we synthesized Fe_3_O_4_-Ce6-FA nanoparticles for FA receptor-targeted PDT. PS and FA were covalently bound to the surface of the magnetite nanoparticles. The prepared multifunctional Fe_3_O_4_-Ce6-FA nanocomposites had high water solubility and good biocompatibility without any cytotoxicity. Moreover, Fe_3_O_4_-Ce6-FA exhibited more effective anticancer activity via apoptosis in prostate (PC-3) and breast cancer (MCF-7) cell lines in comparison with Fe_3_O_4_ conjugated with HPs. Thus, the PS (Ce6) used in this study is more useful for PDT applications owing to its ability to produce a high singlet oxygen quantum yield and deep penetration owing to its long-wavelength absorption band. However, further in vivo studies are required to verify its biological effects for clinical applications, although we believe that our study makes a significant contribution to PDT because it supports the use of chlorin e6 as a PS in multifunctional nanomaterials for effective PTD.

## Abbreviations

CCK-8	Cell Counting Kit-8
Ce6	Chlorin e6
DPBF	1,3-Diphenylisobenzofuran
DPBS	Dulbecco’s phosphate-buffered saline
FA	Folic acid
FCF	Fe_3_O_4_-Ce6-FA
FE-SEM	Field emission scanning electron microscopy
FITC	Fluorescein isothiocyanate
HPs	Hematoporphyrins
LED	Light-emitting diode
Ms	Saturation magnetization
PDT	Photodynamic therapy
PL	Photoluminescence
PLE	Photoluminescence excitation
PS	Photosensitizer
ROS	Reactive oxygen species
TEM	Transmission electron microscopy
XRD	X-ray diffraction

## References

[1] Ferlay J., Soerjomataram I., Dikshit R., Eser S., Mathers C., Rebelo M., Parkin D.M., Forman D., Bray F. (2015). Cancer incidence and mortality worldwide: Sources, methods and major patterns in GLOBOCAN 2012. Int. J. Cancer.

[2] Wang Y., Shim M.S., Levinson N.S., Sung H.-W., Xia Y. (2014). Stimuli-responsive materials for controlled release of theranostic agents. Adv. Funct. Mater..

[3] Tian G., Zheng X., Zhang X., Yin W., Yu J., Wang D., Zhang Z., Yang X., Gu Z., Zhao Y. (2015). TPGS-stabilized NaYbF_4_: Er upconversion nanoparticles for dual-modal fluorescent/CT imaging and anticancer drug delivery to overcome multi-drug resistance. Biomaterials.

[4] Patel N.R., Pattni B.S., Abouzeid A.H., Torchilin V.P. (2013). Nanopreparations to overcome multidrug resistance in cancer. Adv. Drug Deliv. Rev..

[5] Song G., Wang Q., Wang Y., Lv G., Li C., Zou R., Chen Z., Qin Z., Huo K., Hu R. (2013). A low-toxic multifunctional nanoplatform based on Cu_9_S_5_@mSiO_2_ core-shell nanocomposites: Combining photothermal- and chemotherapies with infrared thermal imaging for cancer treatment. Adv. Funct. Mater..

[6] Lovell J.F., Liu T.W.B., Chen J., Zheng G. (2010). Activatable photosensitizers for imaging and therapy. Chem. Rev..

[7] Celli J.P., Spring B.Q., Rizvi I., Evans C.L., Samkoe K.S., Verma S., Pogue B.W., Hasan T. (2010). Imaging and photodynamic therapy: Mechanisms, monitoring, and optimization. Chem. Rev..

[8] Dolmans D.E., Fukumura D., Jain R.K. (2003). Photodynamic therapy for cancer. Nat. Rev. Cancer.

[9] Ding X., Han B.H. (2015). Metallophthalocyanine-based conjugated microporous polymers as highly efficient photosensitizers for singlet oxygen generation. Angew. Chem. Int. Ed..

[10] Gong H., Dong Z., Liu Y., Yin S., Cheng L., Xi W., Xiang J., Liu K., Li Y., Liu Z. (2014). Engineering of multifunctional nano-micelles for combined photothermal and photodynamic therapy under the guidance of multimodal imaging. Adv. Funct. Mater..

[11] Cakmak Y., Kolemen S., Duman S., Dede Y., Dolen Y., Kilic B., Kostereli Z., Yildirim L.T., Dogan A.L., Guc D. (2011). Designing excited states: Theory-guided access to efficient photosensitizers for photodynamic action. Angew. Chem. Int. Ed..

[12] Agostinis P., Berg K., Cengel K.A., Foster T.H., Girotti A.W., Gollnick S.O. (2011). Photodynamic therapy of cancer: An update. CA Cancer J. Clin..

[13] Luo T., Zhang Q., Lu Q.-B. (2017). Photodynamic therapy mediated by indocyanine green with etoposide to treat non-small-cell lung cancer. Cancer.

[14] Mou J., Lin T., Huang F., Shi J., Chen H. (2017). A new green titania with enhanced NIR absorption for mitochondria-targeted cancer therapy. Theranostics.

[15] Ge J., Lan M., Zhou B., Liu W., Guo L., Wang H., Jia Q., Niu G., Huang X., Zhou H. (2014). A graphene quantum dot potodynamic therapy agent with high singlet oxygen generation. Nat. Commun..

[16] Yogo T., Urano Y., Ishitsuka Y., Maniwa F., Nagano T. (2005). Highly efficient and photostable photosensitizer based on bodipy chromophore. J. Am. Chem. Soc..

[17] Reindl S., Penzkofer A., Gong S.-H., Landthaler M., Szeimies R.M., Abels C., Bãumler W. (1997). Quantum yield of triplet formation for indocyanine green. J. Photochem. Photobiol. A.

[18] Tang C.-Y., Wu F.-Y., Yang M.-K., Guo Y.-M., Lu G.-H., Yang Y.-H. (2016). A classic near-infrared probe indocyanine green for detecting singlet oxygen. Int. J. Mol. Sci..

[19] Zhen Z., Tang W., Chuang Y.J., Todd T., Zhang W., Lin X. (2014). Tumor vasculature targeted photodynamic therapy for enhanced delivery of nanoparticles. ACS Nano.

[20] Bechet D., Couleaud P., Frochot C., Viriot M., Guillemin F., Barberi-Heyob M. (2008). Nanoparticles as vehicles for delivery of photodynamic therapy agents. Trends Biotechnol..

[21] Richter A.M., Waterfield E., Jain A.K., Canaan A.J., Allison B.A., Levy J.G. (1993). Liposomal delivery of a photosensitizer, benzoporphyrin derivative monoacid ring A (BPD), to tumor tissue in a mouse tumor model. Photochem. Photobiol..

[22] Wang C., Xu H., Liang C., Liu Y., Li Z., Yang G., Cheng H., Li Y., Liu Z. (2013). Iron oxide @ polypyrrole nanoparticles as a multifunctional drug carrier for remotely controlled cancer therapy with synergistic antitumor effect. ACS Nano.

[23] Wang Z., Chen Z., Liu Z., Shi P., Dong K., Ju E., Ren J., Qu X. (2014). A multi-stimuli responsive gold nanocage-hyaluronic platform for targeted photothermal and chemotherapy. Biomaterials.

[24] Wang Y., Wang K., Zhao J., Liu X., Bu J., Yan X., Huang R. (2013). Multifunctional mesoporous silica-coated graphene nanosheet used for chemo-photothermal synergistic targeted therapy of glioma. J. Am. Chem. Soc..

[25] Zhang C., Zhao K., Bu W., Ni D., Liu Y., Feng J., Shi J. (2015). Marriage of scintillator and semiconductor for synchronous radiotherapy and deep photodynamic therapy with diminished oxygen dependence. Angew. Chem. Int. Ed..

[26] Elsabahy M., Heo G.S., Lim S.-M., Sun G., Wooley K.L. (2015). Polymeric nanostructures for imaging and therapy. Chem. Rev..

[27] Choi K.-H., Wang K.-K., Oh S.-L., Im J.-E., Kim B.-J., Park J.-C., Choi D., Kim H.-K., Kim Y.-R. (2010). Singlet oxygen generating nanolayer coatings on NiTi alloy for photodynamic application. Surf. Coat. Technol..

[28] Choi K.-H., Nam K.C., Kim U.-H., Cho G., Jung J.-S., Park B.J. (2017). Optimized photodynamic therapy with multifunctional cobalt magnetic nanoparticles. Nanomaterials.

[29] Choi K.-H., Nam K.C., Malkinski L., Choi E.H., Jung J.-S., Park B.J. (2016). Size-dependent photodynamic anticancer activity of biocompatible multifunctional magnetic submicron particles in prostate cancer cells. Molecules.

[30] Nam K.C., Choi K.-H., Lee K.-D., Kim J.H., Jung J.-S., Park B.J. (2016). Particle size dependent photodynamic anticancer activity of hematoporphyrin-conjugated Fe_3_O_4_ particles. J. Nanomater..

[31] Choi K.H., Nam K.C., Kim H.J., Min J., Uhm H.S., Choi E.H., Park B.J. (2014). Synthesis and characterization of photo-functional magnetic nanoparticles (Fe_3_O_4_@HP) for applications in photodynamic cancer therapy. J. Korean Phys. Soc..

[32] Park B.J., Choi K.H., Nam K.C., Ali A., Min J.E., Son H., Uhm H.S., Kim H.J., Jung J.S., Choi E.H. (2015). Photodynamic anticancer activities of multifunctional cobalt ferrite nanoparticles in various cancer cells. J. Biomed. Nanotechnol..

[33] Choi K.H., Choi E.W., Min J.E., Son H., Uhm H.S., Choi E.H., Park B.J., Jung J.S. (2014). Comparison study on photodynamic anticancer activity of multifunctional magnetic particles by formation of cations. IEEE Trans. Magn..

[34] Paul S., Heng W.S., Chan L.W. (2013). Optimization in solvent selection for chlorin e6 in photodynamic therapy. J. Fluoresc..

[35] Mathai S., Smith T.A., Ghiggino K.P. (2007). Singlet oxygen quantum yields of potential porphyrin-based photosensitisers for photodynamic therapy. Photochem. Photobiol. Sci..

[36] Rossi L.M., Silva P.R., Vono L.L.R., Fernandes A.U., Tada D.B., Baptista M.S. (2008). Protoporphyrin IX nanoparticle carrier: Preparation, optical properties, and singlet oxygen generation. Langmuir.

